# Fucosylation enhances activity of cytotoxic T lymphocytes against breast cancer

**DOI:** 10.1186/2051-1426-3-S2-P2

**Published:** 2015-11-04

**Authors:** Na Qiao, Elizabeth A Mittendorf, Mao Zhang, Pariya Sukhumalchandra, Jodie L Polan, Samantha Neal, Mikael Rauf, Victor Gall, Anne V Philips, Elizabeth J Shpall, Gheath Alatrash

**Affiliations:** 1 The University of Texas MD Anderson Cancer Center, Houston, TX, USA

## Introduction

Fucosylation is a process by which fucose sugar groups are added to cell surface receptors. This process is mediated by fucosyl transferases that attach terminal fucose groups to acceptor molecules on the cell surface. Fucosylation of cord blood stem cells and human regulatory T cells (Treg) were shown to enhance cord blood engraftment and Treg homing to inflamed tissues. Since tumor tissues have a component of inflammation, we hypothesized that fucosylation of antigen specific cytotoxic T lymphocytes (CTL) ex vivo will enhance their migration into tumors and subsequent killing of tumor cells. Specifically we tested whether ex vivo fucosylation of CTL that target the HLA-A2 restricted HER2/neu-derived peptide, E75, enhances E75-CTL migration and cytotoxic functions.

## Methods

E75-CTL were generated using standard methodologies. Fucosylation was achieved by co-culturing T cells with FTVII enzyme (Targazyme) and GDP fucose. To study migration, fucosylated and non-fucosylated E75-CTL were passed through chambers that were coated with a HUVEC barrier and migrated CTL were detected using cell fluorescence. To examine CTL surface markers, cells were stained for standard co-stimulatory and adhesion molecules and were analyzed using flow cytometry. Calcein AM cytotoxicity assays were used to determine the effects of fucosylation on CTL killing of target cells. For *in vivo* assessment of fucosylation on activity of CTL, NSG mice were engrafted orthotopically with SKBR3-A2 human breast cancer cells and were treated with intravenous injections of 10^6^ fucosylated or non-fucosylated E75-CTL. Mice were examined twice weekly and were sacrificed on week 5 for tissue analysis.

## Results

Fucosylated E75-CTL showed 1.6-fold higher migration through the HUVEC cell barrier compared to non-fucosylated E75-CTL (MFI=14,264 vs. 8,926). Analysis of T cell surface expression of chemokine/adhesion molecules showed approximately a 5-fold increase in CD49d and CD195, and 50% increase in CXCR1 and CXCR3 following fucosylation. Fucosylation enhanced the cytotoxicity of E75-CTL against E75-pulsed T2 targets (cytotoxicity=33% vs. 25%). *In vivo* experiments using SKBR3-A2 breast cancer xenografts showed that fucosylated E75-CTL reduced SKBR3-A2 tumor size by 20%, in comparison with 5% reduction in the tumor size by non-fucosylated E75-CTL. Lastly, flow cytometry showed a 2-fold increase in the frequency of human CD45+ lymphocytes in the primary tumor following fucosylation.

## Conclusions

Fucosylation enhances the migration and cytotoxic functions of E75-CTL. Our data demonstrate a novel approach to enhance the efficacy of antigen specific T cells that could be used in adoptive cellular immunotherapy approaches for breast cancer.

